# Image-based assessment of tail docking and tail biting in slaughtered pigs across three European countries

**DOI:** 10.3389/fvets.2026.1751411

**Published:** 2026-01-30

**Authors:** Anastasia Romano, Simona Baghini, Andrea Capobianco Dondona, Nicola Bernabò, Giuseppe Marruchella

**Affiliations:** 1 Department of Veterinary Medicine, University of Teramo, Teramo, Italy; 2 Farm4Trade S.r.l., Chieti, Italy; 3 Department of Bioscience and Technology for Food, Agriculture and Environment, University of Teramo, Teramo, Italy

**Keywords:** abattoir, digital images, pig, scoring methods, tail-biting, tail-docking

## Abstract

Slaughterhouse monitoring provides a cost-effective and suitable tool for large-scale surveillance of tail-biting, which is a major welfare issue in pig production. The European Union Council Directive 2008/120/EC prohibits routine tail-docking as a preventive measure against tail-biting. Nevertheless, compliance remains inconsistent, and tail-docking is still widely practiced in Europe. This study aimed to assess the occurrence of tail-biting and tail-docking in slaughtered pigs (*n* = 15,000) from Italy, Netherlands and Spain using digital images. Results indicate that most pigs were tail-docked (88.1%), with substantial variation among countries: tail-docking was most common in Spain (99.4%), followed by Netherlands (86.5%), and least common in Italy (78.5%). Overall, tail-biting lesions were observed in 5.4% of pigs, with the highest prevalence in Italy (11.6%), followed by Netherlands (3.4%), and Spain (1%). The differences among the three countries were significant (*p* < 0.0001), tail lesions being more frequent in pigs with undocked tails than docked tails (*p* < 0.0001). The risk of having a lesion was substantially higher in pigs with undocked/intermediate tails (relative risk = 4.6). The severity of lesions was scored using two different methods, which showed an almost perfect agreement (weighted Cohen’s kappa coefficient 0.826; *p* < 0.0001). Lesions were most frequently detectable in the two lateral views, whereas the central view alone was inconclusive in most of pigs (99%).

## 1 Introduction

Tail-biting refers to a range of detrimental behaviors expressed by domestic pigs, and according to Taylor *et al*. (1) it could be properly defined as “any oral manipulation of the tail resulting in lesions.” Although first described in the 19th century, tail-biting became a widespread and severe problem with the development of intensive indoor pig farming, approximately 70 years ago. Nowadays, it is widely regarded as an “iceberg indicator” of impaired animal welfare, as its occurrence often reflects underlying management or health issues. Although it can happen in all production systems including outdoor, tail-biting outbreaks are more commonly reported in indoor pig herds exposed to risk factors such as high stocking density, barren environments (e.g., lack of manipulable materials, poor ventilation), inadequate nutrition, and suboptimal health status (1, 2).

The etiology and pathogenesis of tail-biting is complex, multifactorial, and still debated. Three main clinical forms of tail-biting are currently reported, and distinguishing among them is crucial for effective prevention and control:

a) Two-stage tail-biting – This is considered the most common form and begins with gentle oral manipulation of a pen mate’s tail, without causing overt discomfort (“first stage”). If manipulation progresses to skin damage, bleeding may occur and subsequently trigger more intense biting episodes (“second stage”).
b) Sudden-forceful tail-biting – This form occurs less frequently and onsets abruptly among pigs competing for limited resources, such as feed or water.
c) Obsessive (or fanatical) tail-biting – In this pattern, one or a few pigs persistently seek out and bite the tails of conspecifics, often inflicting severe lesions irrespective of resource availability or environmental conditions (1, 3).

Because biting arises from farm-specific combinations of risk factors, tail lesions do not reflect the identical welfare concerns across all studies. In some contexts, they are primarily associated with environmental or resource limitations and behavioral frustration, whereas in others they relate more to climatic stress, health status, or management practices. Such context dependence likely contributes to seemingly contradictory findings, even though tail-biting is generally regarded as an indicator of elevated welfare risk (1–4).

Tail-biting has a substantial negative impact on the profitability of pig production, as it is associated with reduced growth rates and feed efficiency, increased labor and veterinary costs, and a higher prevalence of carcass condemnations due to abscesses and arthritis (4, 5). Tail-docking – i.e., the partial amputation of piglets’ tails shortly after birth – has long been the most widespread preventive measure (6, 7). However, tail-docking itself induces acute pain and may lead to long-term hypersensitivity, making it a significant welfare concern (8, 9).

The European Union (EU) Council Directive 2008/120/EC states that *“neither tail-docking nor reduction of corner teeth must be carried out routinely, but only where there is evidence that injuries to sows’ teats or to other pigs’ ears or tails have occurred. Before carrying out these procedures, other measures shall be taken to prevent tail biting and other vices, taking into account the environment and stocking densities. For this reason, inadequate environmental conditions or management systems must be changed”* (Annex I, Chapter I, Point 8). Despite this, compliance remains inconsistent, and the procedure is still widely practiced across most EU Member States (5, 10, 11). Currently, tail-docking is strictly forbidden in Finland and Sweden. Outside the EU, fewer than 5% of pigs are tail-docked in Norway and Switzerland, whereas the practice remains allowed and routinely performed in several major pig-producing countries, including the United States, Canada, Brazil, and China (10–12).

This study aimed to assess the occurrence of tail-biting and tail-docking in slaughtered pigs from three EU countries, using digital images collected at the abattoir.

## 2 Materials and methods

### 2.1 Animals

Investigations were carried out in three high-throughput abattoirs located in Italy, Spain, and Netherlands, respectively. A total of 15,000 slaughtered pigs were studied, consisting of:

- 5,000 heavy pigs from Italy (160–180 kg live weight; 9–10 months old).
- 5,000 pigs from Netherlands (90–100 kg live weight, 5–6 months old).
- 5,000 pigs from Spain (125–130 kg live weight, 6–7 months old).

The sample size (i.e., 5,000 pigs *per* slaughterhouse) was chosen to provide robust statistical power despite the heterogeneous distribution of expected lesion-class frequencies.

### 2.2 Photo acquisition

**Figure 1:**
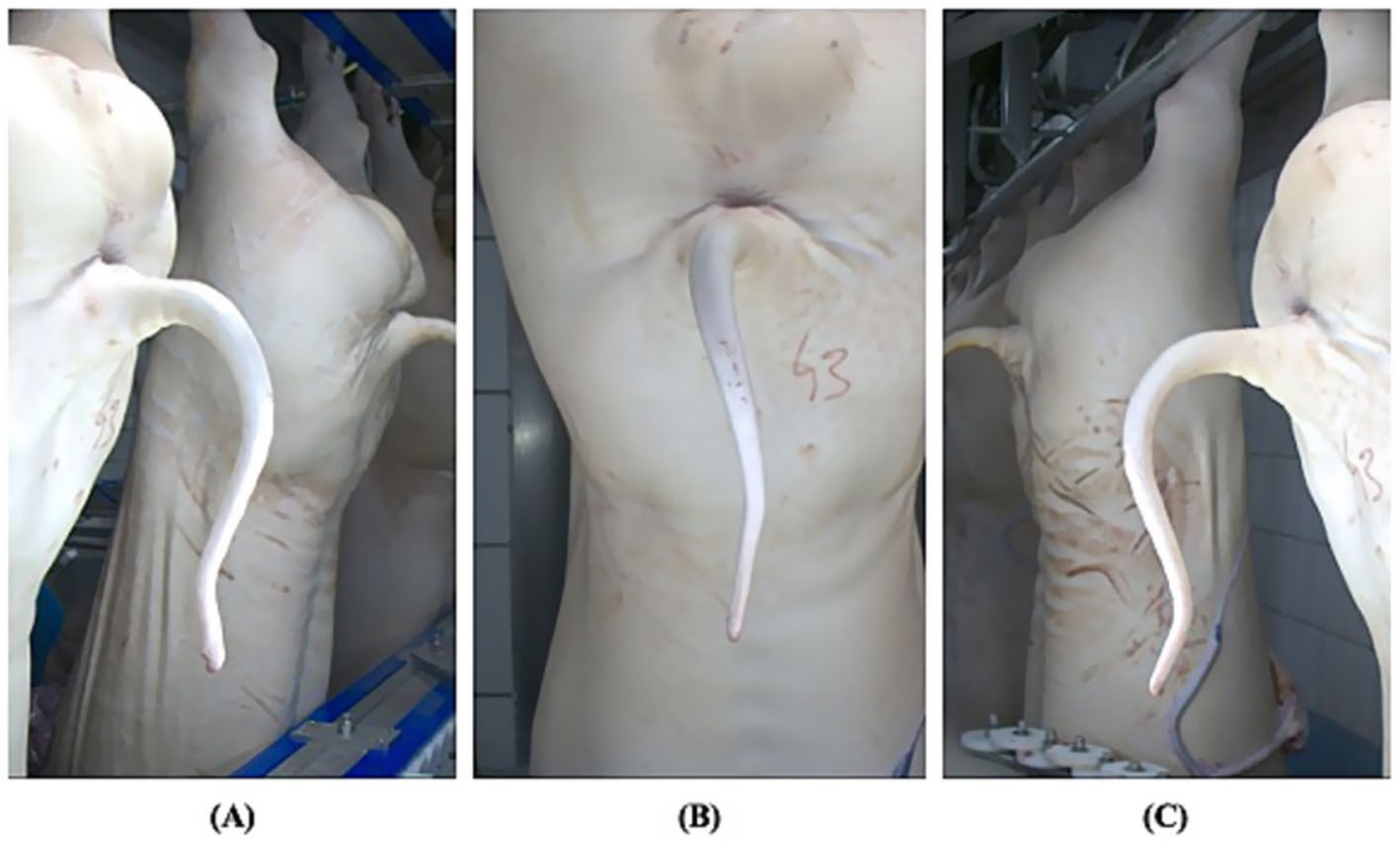
Three pictures of the same tail, taken from the left **(A)**, central **(B)**, and right **(C)** views. A small indentation of the tail tip is clearly visible in the left view **(A)**.

Digital images were captured along the slaughter line – after carcass scalding and dehairing – by means of a collaborative 6-axis robotic arm equipped with two high-resolution cameras (13). More in detail, three images *per* carcass were acquired (right, central, and left views) to ensure optimal visualization of the tail (Figure 1).

### 2.3 Tail-docking and tail-biting assessment

**Figure 2:**
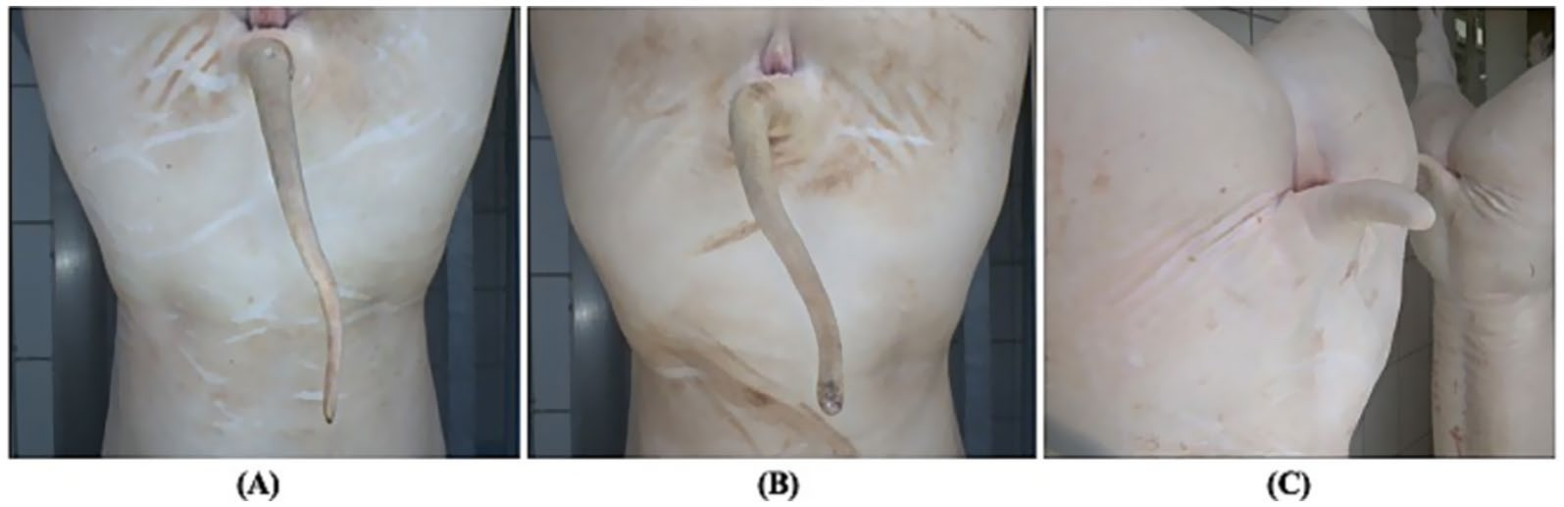
**(A)** Undocked tail, progressively tapering with a typical flat tip. **(B)** Tail of comparable length to many undocked tails, therefore incompatible with tail docking but lacking the characteristic flat tip. In both **(A,B)**, the brownish appearance of the tail tip is due to the slaughtering process (burning of residual epidermis after scalding and brushing). **(C)** Docked tail, markedly shorter than in **(A,B)**, with a thicker, rounded tip and no lesions due to tail-biting.

Images were listed by abattoir and slaughter day, and subsequently uploaded to a dedicated online platform, which enabled the recording of the following features:

a) Tail-docking status – based on tail length and shape, pigs were classified as: (i) undocked, i.e., intact tail displaying the typical flat tip; (ii) docked, i.e., tail length ≤50% of that of an undocked tail; and (iii) intermediate, i.e., tails similar in length to undocked ones but lacking the flat tip, a condition likely due to tail-biting (Figure 2). Partial and total tail loss were excluded from this classification, as the absence of historical information made it impossible to determine whether such pigs had originally been docked or undocked.
b) Tail-biting lesions – a unique score was assigned to each pig, after the assessment of the three corresponding images. To this aim, two different scoring methods were employed (see Table 1 for details). In addition, the following pathological findings were recorded whenever present: wounds, ulcer/necrosis, fracture/dislocation, total/partial tail loss.

**Table 1:** Scoring methods adopted in this study.

Score	Scoring method A*	Score	Scoring method B**
0	No evidence of tail-biting, regardless of tail length.	0	No evidence of tail biting.
1	Superficial or puncture injuries, with hyperemia and oedema of the wound edges; linear scar (healing by first intention) and/or healed indentation of the tail tip.	1	Healed or mild lesions.
		2	Evidence of chewing or puncture wounds, no swelling.
2	Large wounds, ulcer and/or necrosis, with hyperemia and oedema of the lesion; large and irregular scars (healing by second intention), including healed, partial or total tail loss, and severe deformities.	3	Evidence of chewing or puncture wounds with swelling and signs of infection.
		4	Partial or total loss of the tail.

* This simplified scoring method (herein indicated as “scoring method A”) was developed by the authors, considering both acute and chronic lesions. ** According to Kritas and Morrison (33).

All assessments were performed by three veterinarians (AR, SB, and GM) following multiple joint training sessions performed both at the slaughterhouse and using digital image sets. Prior to study initiation, agreement between direct tail inspections at the slaughterhouse and tail assessments based on digital photographs was evaluated: the prevalence-adjusted bias-adjusted kappa (PABAK) ranged from 0.73 (GM; observed agreement: 86.5%, based on 1,518 pigs) to 0.78 (AR; observed agreement: 89.3%, based on 619 pigs). In addition, veterinarians (namely AR and GM) achieved substantial agreement during both direct tail inspection at the slaughterhouse (observed agreement: 86.9%, PABAK = 0.74, based on 2,677 pigs) and tail assessment on digital images (observed agreement: 89.5%, PABAK = 0.79, based on 1,614 pigs).

Image included in this study were jointly evaluated by at least two investigators, who reached consensus on the final rating.

The data associated with each triplet of images (i.e., one pig) did not permit tracing back to the farm of origin. Therefore, slaughter days were randomly selected (30-to-40 days *per* abattoir, from May 2024 to September 2025). Assessments were consistently carried out on sequences of 40 consecutively slaughtered pigs, randomly selected within each slaughter day and likely representing a single batch. Finally, investigators recorded whether lesions were visible in the right, and/or central, and/or left image.

### 2.4 Statistical analysis

Data were recorded in a Microsoft® Excel spreadsheet, and subsequently analyzed to assess: (a) the proportion of tails that were docked (overall and abattoir-specific), with differences among countries evaluated using the chi-square test; (b) the prevalence and severity of tail-biting lesions (overall and abattoir-specific), with differences among countries evaluated using the chi-square test; (c) the prevalence of different pathological findings (overall and abattoir-specific); (d) the association between tail length and biting-related lesions, evaluated using the chi-square test; (e) the relative risk of having tail-biting lesions in pigs with undocked/intermediate *vs*. docked tail; (f) the agreement between the two scoring methods, assessed using the weighted Cohen’s kappa coefficient; and (g) the randomness of lesions and undocked/intermediate tails, evaluated through the non-parametric Wald-Wolfowitz test.

## 3 Results

### 3.1 Tail-docking in slaughtered pigs

Overall, 13,210 out of the 15,000 investigated pigs (88.1%) were classified as tail docked. More in detail, the proportion of tail-docked pigs differed among countries, amounting to 78.5% in Italy, 86.5% in Netherlands, and 99.4% in Spain, differences being statistically significant (χ^2^ = 1063.28; *p* < 0.0001).

The proportion of undocked/intermediate tails was 10.04% overall (1,506 pigs), amounting to 17.4% in Italy, 12.82% in Netherlands, and 0.12% in Spain. As detailed above, pigs exhibiting total or partial tail loss were excluded from this count.

The distribution of undocked/intermediate tails showed a clear clustering pattern, which was markedly stronger in Netherlands than in Italy (Z value 62.18 and 19.40, respectively; *p* < 0.0001).

### 3.2 Tail-biting lesions in slaughtered pigs

Overall, biting-related tail lesions were detected in 803 slaughtered pigs (5.4%). The prevalence of lesions was 11.6% in Italy, 3.5% in Netherlands, and 1% in Spain. The difference among the three countries was highly significant (χ^2^ = 502.84; *p* < 0.0001). Mild lesions (i.e., score 1 of the method A, proposed herein) were recorded in 312 pigs, whereas severe lesions (i.e., score 2) were observed in 491 pigs (see Table 2 for details).

**Table 2:** Main features of tail-biting.

Tail lesions	Overall	Italy*	Netherlands*	Spain*
Tail-biting lesions	803	582 (72.5%)	172 (21.4%)	49 (6.1%)
Mild lesions	312	236 (75.6%)	70 (22.4%)	6 (1.9%)
Severe lesions	491	346 (70.5%)	102 (20.8%)	43 (8.7%)
Pathological findings				
Wounds	104	55 (52.9%)	40 (38.5%)	9 (8.6%)
Ulcer/necrosis	72	19 (26.4%)	28 (38.9%)	25 (34.7%)
Scars	480	400 (83.3%)	63 (13.1%)	17 (3.5%)
Fractures/dislocations	36	33 (91.7%)	2 (5.5%)	1 (2.8%)
Partial or total tail loss	273	214 (78.4%)	34 (12.4%)	25 (9.1%)

* Percentage values were calculated with respect to the total number of each type of lesion. Multiple lesions may have been observed on the same tail.

A significant association was found between tail length and the presence of tail-biting lesions; pigs with undocked or intermediate tails showed a markedly higher prevalence of lesions compared to those with docked tails (χ^2^ = 486.80; *p* < 0.0001). Moreover, the risk of tail-biting lesion was substantially higher in pigs with undocked/intermediate tails (relative risk = 4.6).

The agreement between the two scoring methods was positive and very strong (weighted Cohen’s kappa coefficient 0.826; *p* < 0.0001). All mild lesions were assigned a score of 1 using both scoring systems. In addition, 433 out of 493 lesions were consistently classified as severe using both the method A proposed herein (score 2) and the system developed by Kritas and Morrison (101 pigs scored 3 and 332 pigs scored 4). Tail lesions showed a strong and significant tendency to cluster (Z value of −6.86; *p* < 0.0001).

In 53% of cases, the lesion was clearly visible in all three images acquired for each pig. In 35% of cases, it was detected in one (28%) or both (7%) lateral views, whereas in 11% it was evident in the central view and in a single lateral view. The lesion was visible exclusively in the central image in only 1% of cases.

The results of all statistical analyses are summarized in Table 3.

**Table 3:** Summary of statistical analyses and relationships between tail lesions, tail length, and image-based assessment.

Outcome/relationship	Analyzed variables	Statistical test	Main findings*
Tail-docking prevalence	Country (Italy, Netherlands, Spain)	Chi-square test	The proportion of tail-docked pigs differed significantly among countries, being highest in Spain and lowest in Italy
Prevalence of tail-biting lesions	Country (Italy, Netherlands, Spain)	Chi-square test	Tail-biting lesions were significantly more frequent in Italy than in Netherlands and Spain
Association between tail length and tail-biting lesions	Tail length (docked *vs.* undocked/intermediate)	Chi-square test	Tail-biting lesions were significantly more prevalent in pigs with undocked/intermediate tails
Risk of tail-biting lesions	Tail length (docked *vs.* undocked/intermediate)	Relative risk	Pigs with undocked/intermediate tails showed a markedly higher risk of tail-biting lesions
Agreement between scoring methods	Scoring method A *vs.* scoring method B	Weighted Cohen’s kappa coefficient	The agreement between the two scoring systems was almost perfect
Distribution of undocked/intermediate tails	Sequential carcasses within slaughter batches	Wald–Wolfowitz runs test	Undocked/intermediate tails showed significant clustering, particularly in Netherlands
Spatial distribution of tail-biting lesions	Sequential carcasses within slaughter batches	Wald–Wolfowitz runs test	Tail-biting lesions showed significant clustering

* All reported differences were statistically significant (*p* < 0.0001). The agreement between scoring methods was interpreted according to standard criteria for Cohen’s kappa coefficient.

## 4 Discussion

As recently emphasized by D’Alessio *et al*. (14), collecting reliable data on the prevalence of tail-biting and tail-docking in EU countries is essential. In this context, the Directorate General for Health and Food Safety of the European Commission recommend the assessment of pig tails at the slaughterhouse, by recording the prevalence and severity of intact and bitten tails for benchmarking purposes (15).

Although the lesions assessment at the abattoir is affected by intrinsic biases (e.g., slaughter procedures) and is mainly suitable for detecting chronic lesions compatible with animal survival (16, 17), both EU legislation [Regulation 625/2017; (18)] and EFSA scientific opinion (19) recognize slaughterhouses as key settings for monitoring pig health and welfare, whether it can be done under standardized conditions (20–22).

This study confirms that tail-docking remains a common practice in Italy, Netherlands, and Spain, although notable differences emerged among these countries. In particular, the proportion of docked pigs approached 100% in Spain, was slightly below 80% in Italy, and reached an intermediate value in Netherlands. It would be highly informative to trace back the farms of origin, as the clustering of pigs with tail-biting lesions or undocked/intermediate tails suggests a relevant batch-related effect. Accordingly, the prevalence of tail lesions at slaughter largely reflects conditions during the rearing and finishing, as consistently shown across studies, with the farm of origin playing a key explanatory role (5, 10, 19).

Considering the Italian context, it is worth noting that all pigs included in this study were born and reared in Italy, where a national programme is currently in place requiring that 15% of piglets be raised without tail docking (data available at https://www.anmvioggi.it/images/ITER_OPERATIVO_PIANO_TAGLIO_CODA_copy.pdf, last accessed on 20th November 2025). This proportion is intended to be progressively increased provided that no major issues are detected. The data reported herein appear consistent with the ongoing implementation of this programme.

Prevalence estimates of tail-biting lesions in slaughtered pigs vary widely across studies, due to methodological differences: (a) the scoring system applied; (b) the prevalence of tail-docking in the population; (c) the definition of an intact tail; (d) sample size; and (e) investigative approach, i.e., routine postmortem monitoring *vs*. targeted experimental studies (5, 23–25). This hampers the comparability of currently available data and underscores the need for standardized methods to assess and record tail lesions at the abattoir. In this study, the overall prevalence of tail lesions was 5.35%, with significant differences among countries. According to previous studies (26–32), tail-biting prevalence was strongly associated with undocked tails, which likely explains the higher proportion of lesions observed in Italy. However, differences in on-farm conditions among Spain, Italy, and Netherlands may affect the prevalence and severity of tail lesions and should also be considered when interpreting such data.

At present, the European Union does not recommend or mandate any official or harmonized method for the detection and scoring of tail lesions in slaughtered pigs. As a rule, scoring systems should be rapid, simple, and easily standardized, to ensure suitability for high-throughput abattoirs. In our opinion, the method proposed herein satisfactorily meets such criteria. Moreover, it agrees strongly with the more complex and widely used method developed by Kritas and Morrison (33). Detailed classification of pathological findings provides valuable insights for data interpretation and facilitates targeted mitigation strategies. Overall, severe lesions were more frequently detected, likely because they are easier to recognize (14). The prevalence of scars in Italy was much higher than that reported in Spain and Netherlands, which is reasonably due to the older slaughter age of Italian pigs (9–10 months vs. 5–7 months). This factor should be carefully considered, as healed or chronic lesions are inherently more difficult to identify (32).

As demonstrated by D’Alessio *et al*. (14), visual-only scoring of tail lesions is a reliable alternative to direct inspection along the slaughter line. Moreover, image-based assessment offers greater flexibility in scheduling, allows more precise evaluation, and provides a robust basis for automation of the entire scoring process. Initially, three images *per* carcass were considered necessary to adequately cover the tail surface. However, our results indicate that the central view alone revealed lesions in a negligible proportion of pigs. Thus, acquiring three images appears redundant, and the use of two lateral views is recommended, in line with the findings of Vom Brocke *et al*. (24) and Brünger *et al*. (20). This point is particularly relevant for high-throughput slaughterhouses, as it streamlines data acquisition and processing, lowers algorithmic complexity, and reduces time and cost demands without compromising assessment performance.

The automated acquisition of suitable pictures, image-based assessment of tail lesions, and batch-level traceability constitute essential prerequisites for the full automation of the scoring process (14, 20, 24, 34). Such automation is highly desirable, as it would enable large-scale data collection, allow identification of farms with an elevated risk of underlying welfare problems, improve the estimation of farm-specific risk factors, and further strengthen the role of tail-biting as an indicator of on-farm welfare conditions.

In conclusion, this study confirms that tail-docking is still widely practiced in European Union member states, although in different proportions among countries, and that tail-biting lesions are more frequent in pigs with undocked tails. Healed and chronic lesions are by far the most common findings at slaughter, particularly in Italian heavy pigs. This must be taken into account when developing and applying scoring systems tailored for slaughter pigs (32). Finally, collecting two images *per* carcass appears sufficient to assess the presence of tail lesions. This agrees with the literature (20, 24) and should be duly considered in view of future automation of scoring of tail-biting lesions.

## Data Availability

The original contributions presented in the study are included in the article/supplementary material, further inquiries can be directed to the corresponding author.
